# Prediction values of fat-soluble vitamin of growth retardation in children aged 1–6 years

**DOI:** 10.3389/fped.2024.1315115

**Published:** 2024-10-21

**Authors:** Qingqing Yan, Qingwen Zhu, Chen Jiang, Lingli Zhang, Xiaojing Xu

**Affiliations:** ^1^ Department of Prenatal Screening and Diagnosis Center, Affiliated Maternity and Child Health Care Hospital of Nantong University, Nantong, Jiangsu, China; ^2^ Center of Laboratory Medicine, Affiliated Hospital of Nantong University, Nantong, Jiangsu, China; ^3^ Department of Children's Health Center, Affiliated Maternity and Child Health Care Hospital of Nantong University, Nantong, Jiangsu, China

**Keywords:** growth retardation, children, fat-soluble vitamins, vitamin deficiency, prediction value, retrospective observational study

## Abstract

**Background:**

Retardation among children is a persistent global health concern. Vitamin deficiency in childhood may contribute to growth retardation; however, its causal effects are not fully understood.

**Objective:**

Here, we aimed to explore the prediction values of fat-soluble vitamin levels on GR in children aged 1–6 years.

**Methods:**

614 children aged from 1 to 6 years at Nantong Maternal and Child Care Health Hospital between January 2021 and December 2022 in this retrospective observational study participated in the assessment of developmental status and blood detection of vitamin from peripheral blood (PB). The relationship between vitamin levels and GR was analyzed by Multivariable logistic regression analysis.

**Results:**

Developmental assessment results showed that 132 cases from 614 with growth retardation (21.50%). Statistical analysis showed children with GR were more likely to be males (59.45% vs. 40.55%, *p* = 0.191). The age of children with GR was significantly higher than those without GR (*p* < 0.01). Importantly, the levels of various fat-soluble vitamins in GR individuals were significantly lower than those in normal individuals. ROC analysis showed that vitamin E, vitamin A and 25(OH) D_3_ were less effective in predicting GR model (AUC: 0.87, 0.74, and 0.65,). However, the combination of vitamin E, vitamin A and 25(OH)D_3_ with age was effective in predicting GR. (AUC: 0.84, 0.77, 0.75).

**Conclusion:**

The combination of 25(OH)D3, Vitamin E, Vitamin A with age may have good predictive performance for children GR aged 1–6 years.

## Introduction

Growth retardation (GR) characterized by short stature, low weight, and distinctive facial features is a prevalent disease among children (1). GR is divided into intrauterine growth retardation (IUGR) and extrauterine growth retardation (EUGR). IUGR is defined as a birth weight of newborns below the 10th percentile or two standard deviations of the average weight for the same age (2). While, EUGR defines the growth and development of a newborn after birth, meaning that its growth indicators at all stages are consistently below the 10th percentile level of the expected value (3). Research indicates that IUGR is closely linked to genetic factors, individual variability, and nutritional status both pre-pregnancy and during pregnancy (4). Conversely, EUGR is more significantly influenced by postnatal factors, encompassing both heredity and nutrition (5, 6). While the prevention of EUGR may seem relatively more straightforward than that of IUGR, the multifaceted nature of its influencing factors (including age, nutritional levels, gender et al.,) can lead to misdiagnosis in the professional assessment of GR in children. To truly control childhood GR, integrating various factors into a precise predictive model to develop an effective prevention strategy urgent and necessary.

Despite significant advancements in neonatal care and treatment, the incidence of GR has shown an increasing trend in recent years. In 2010, a staggering 56.70% of children under the age of five (approximately 171.4 million) were suffered from GR worldwide. The prevalence of GR is especially high in regions such as South Asia, Central Asia, and sub-Saharan Africa 0 (7). For instance, in Bangladesh, 36% of children under five years old suffer from stunted growth (8), Meanwhile, in some parts of China, the incidence of GR has climbed to 7.84%. Collectively, GR of children remains a significant global health challenge (9, 10).

Soliman (11) and Verma (12) asserted that chronic nutritional deficiencies, rooted in inadequate long-term nutrient intake, significantly contribute to stunted growth—a viewpoint also endorsed by Black (13, 14). In 2019, to address the global issue of children GR, the World Health Organization (WHO) prioritized enhancing maternal and child nutrition management (15, 16). Fat-soluble vitamins, indispensable for human health, play pivotal roles in growth, development, and immune regulation. The intake disparities of fat-soluble vitamins, whether insufficiency or excess, can result in GR or other diseases. Current research underscores the critical role of vitamins A, D, and E in the embryonic development of bones, muscles, and the nervous system. Supplementing with vitamins D3 and K can facilitate bone development, and sustains calcium homeostasis. Chen C's (17) study indicated that elevated vitamin D levels in late pregnancy may mitigate the risk of GR in children. Despite the involvement of most fat-soluble vitamins in growth regulation and metabolism, the causal link between these vitamins and growth retardation is yet to be conclusively established. Nonetheless, considering the functions of fat-soluble vitamins, it is plausible to hypothesize a correlation between their levels and the incidence of GR.

In this study, we performed developmental assessment and detection of vitamin in peripheral blood in children aged 1–6 years, which aimed to establish a model for vitamin deficiency to predict GR in children by regression analysis.

## Methods

### Ethics statement

This study was approved by the Ethics Committee of Nantong Maternal and Child Care Health Hospital Affiliated to Nantong University, China. The parents of the children were informed about this study and provided informed consent (Y2020088).

### Sample collection

This retrospective observational study enrolled children aged 1–6 years at the Nantong Maternal and Child Care Health Hospital Affiliated to Nantong University, China, between January 2021 and December 2022. Data were extracted from the medical records of the patients, including sex and age.

### Study design and sample selection

The inclusion criteria were as follows: (1) participants aged 1–6 years old; (2) GR diagnosis confirmed by clinical, laboratory, and imaging examinations; (3) no systemic medication and psychotherapy administered for GR.

The exclusion criteria were as follows: (1) A history of premature birth, low birth weight, or intrauterine growth restriction; (2) congenital or acquired organ or tissue deficiency disease; (3) presence of respiratory, digestive, urinary, blood, or endocrine factors that may affect growth and development; (4) mental or nervous system diseases resulting in hearing and speech difficulties or inability to cooperate with the study; (5) hereditary growth retardation; (6) incomplete clinical data.

### Vitamin detection in peripheral blood by LC-MS/MS

Peripheral blood samples (3 ml) of each participant for vitamin detection were collected prior to eating on the day of the test. The concentrations of Vitamin A, 25-Dihydroxy vitamin D2 [25(OH)D2], 25(OH)D3, Vitamin E and Vitamin K1 were determined using liquid chromatography-mass spectroscopy/mass spectroscopy (LC-MS/MS) analysis by Ultra-high-performance liquid chromatography (Waters Acquisition, USA) and triple four-bar mass spectrometry (XEVO TQD, USA).

### Bioinformatic analysis

The Chi-square test was employed to analyze the correlation between various factors and children GR (Table 1); the relationship between vitamins (A, D3, E) and children GR was explored through covariance analysis, with age and gender designated as covariates (Table 3). A binary logistic multivariate analysis was conducted on vitamins (A, D3, E) and age. The Receiver Operating Characteristic (ROC) curve analysis was utilized to determine the Area Under the Curve (AUC) values and to evaluate the predictive values. The Youden Index was applied to ascertain the threshold, with a two-sided *p*-value less than 0.05 indicating statistical significance. The state variable value was set for the normal control group, and the test variables were vitamins (A, D, E) and age (Table 4).

**Table 1:** Comparison of general data.

Variables	Growth retardation	Normal development	*x*2	*p*-value
	(*n* = 132)	(*n* = 482)		
Child factors				
Age (year)				
≥1, ≤3	25 (18.90)	261 (54.10)	51.63	<0.01
>3, ≤6	107 (81.1)	221 (45.9)		
Sex (%)				
Male	85 (64.4)	280 (58.1)	1.71	0.191
Female	47 (35.6)	202 (41.9)		
Local area (%)				
Nantong local area	106 (80.3)	368 (76.3)	0.92	0.337
Not in the local area	26 (19.7)	114 (23.7)		
Parental factors				
Educational level (%)				
Bachelor's degree or above	45 (34.1)	148 (30.7)	0.55	0.458
Below bachelor's degree	87 (65.9)	334 (69.3)		
Mother's gestational age (%)				
35 years old or older	18 (13.6)	78 (16.2)	0.46	0.521
Under 35 years old	114 (86.4)	404 (83.8)		
Pregnancy related diseases (%)				
Pregnancy complicated with diabetes	13 (9.8)	38 (7.9)	0.53	0.469
Normal pregnancy	119 (90.2)	444 (92.1)		
Pregnancy complicated with anemia	10 (7.6)	42 (8.7)	0.17	0.677
Normal pregnancy	122 (92.4)	440 (91.3)		
Pregnancy complicated with thyroid disease	8 (6.1)	46 (9.5)	0.53	0.469
Normal pregnancy	124 (93.9)	436 (90.5)		
Method of conception (%)				
Unnatural conception	3 (2.3)	31 (6.4)	3.43	0.064
Nature conceived	129 (97.7)	451 (93.6)		

### Statistical analysis

The significance of between-group differences was assessed using Covariance analysis with SPSS (version 26.0; SPSS Inc., Chicago, IL, USA). Normally distributed continuous data are presented as the mean ± SD. Skewed distributed continuous data are presented as median (range) and were compared using the Mann-Whitney *U*-test. Categorical data are described as *n* (%) and were compared between groups using the chi-squared test.

## Results

### Overview of the survey

**Figure 1:**
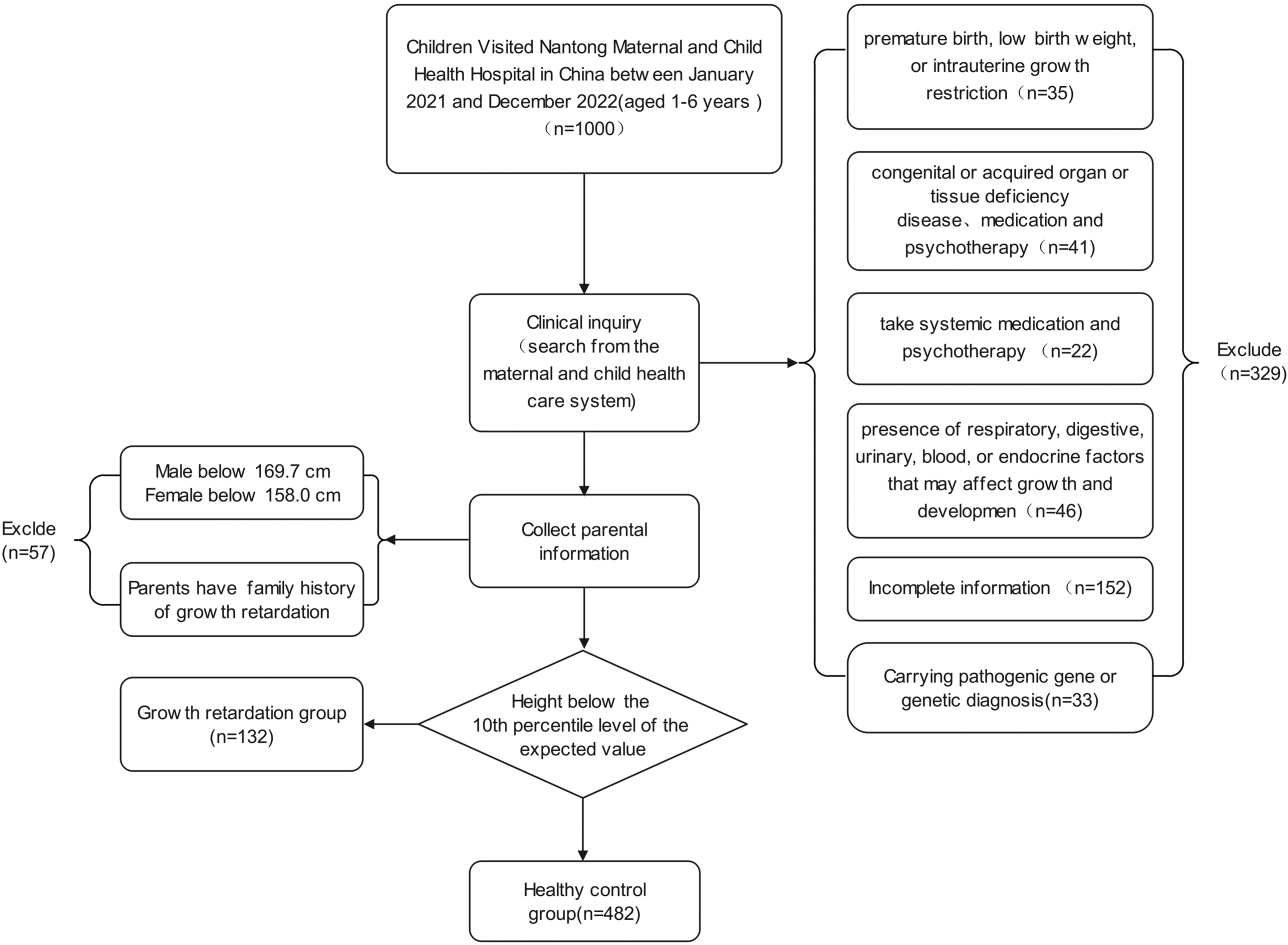
Incorporate into the research flowchart.

614 form 1,000 children aged 1–6 years old were determined to meet the selection criteria. Ultimately, 132 of 614 children were identified as GR based on body condition assessments (Figure 1). The results of statistical analysis found that, children with GR were more likely to be males compared to children without GR (*p* = 0.191). There was also a difference in age between the two groups, which showed that children with GR are older than those without GR. Importantly, in the GR sample, GR was more likely to occur in individuals aged 4–6 years compared to individuals aged 1–3 years (*p* < 0.01). At the same time, we also paid attention to the influence of other factors on children's GR, and the results showed that: the factors such as Local area, Parental factors, Mother's gestational age, Pregnancy related diseases, Mother's gestational age are not the main factors for GR in children (Table 1).

### Vitamin content comparison in children with GR or not

Next, we compared the difference of different fat-soluble vitamins in children with GR or not by controlling Gender and age factors via covariance analysis. The results showed that the level of vitamin A, D and E in children with GR is significantly lower than that in children without GR. For the detail: Vitamin A (230.35 ± 66.01 vs. 289.19 ± 70.28, *p* < 0.01), 25(OH)D3 (23.95 ± 7.30 vs. 27.91 ± 7.91, *p* < 0.01), Vitamin E (4.37 ± 1.77 vs. 7.03 ± 1.92, *p* < 0.01) (Table 2).

**Table 2:** Comparison of vitamin indexes data.

Variables	Growth retardation	Normal development	*F*/*Z*	*p*-value
	(*n* = 132)	(*n* = 482)		
Vitamin A (ng/ml)	230.35 ± 66.01	289.19 ± 70.28	20.196	<0.01
25(OH)D3 (ng/ml)	23.95 ± 7.3	27.91 ± 7.91	16.733	<0.01
Vitamin E (µg/ml)	4.37 ± 1.77	7.03 ± 1.92	61.237	<0.01
25(OH)D2 (ng/ml)	0.30, (0.86,0.62)	0.28, (0.82,0.42)	0.126	0.776
Vitamin K1 (ng/ml)	0.22 (0.84,0.42)	0.41, (1.14,0.70)	−5.043	<0.01

### Multivariable logistic regression analysis

**Figure 2:**
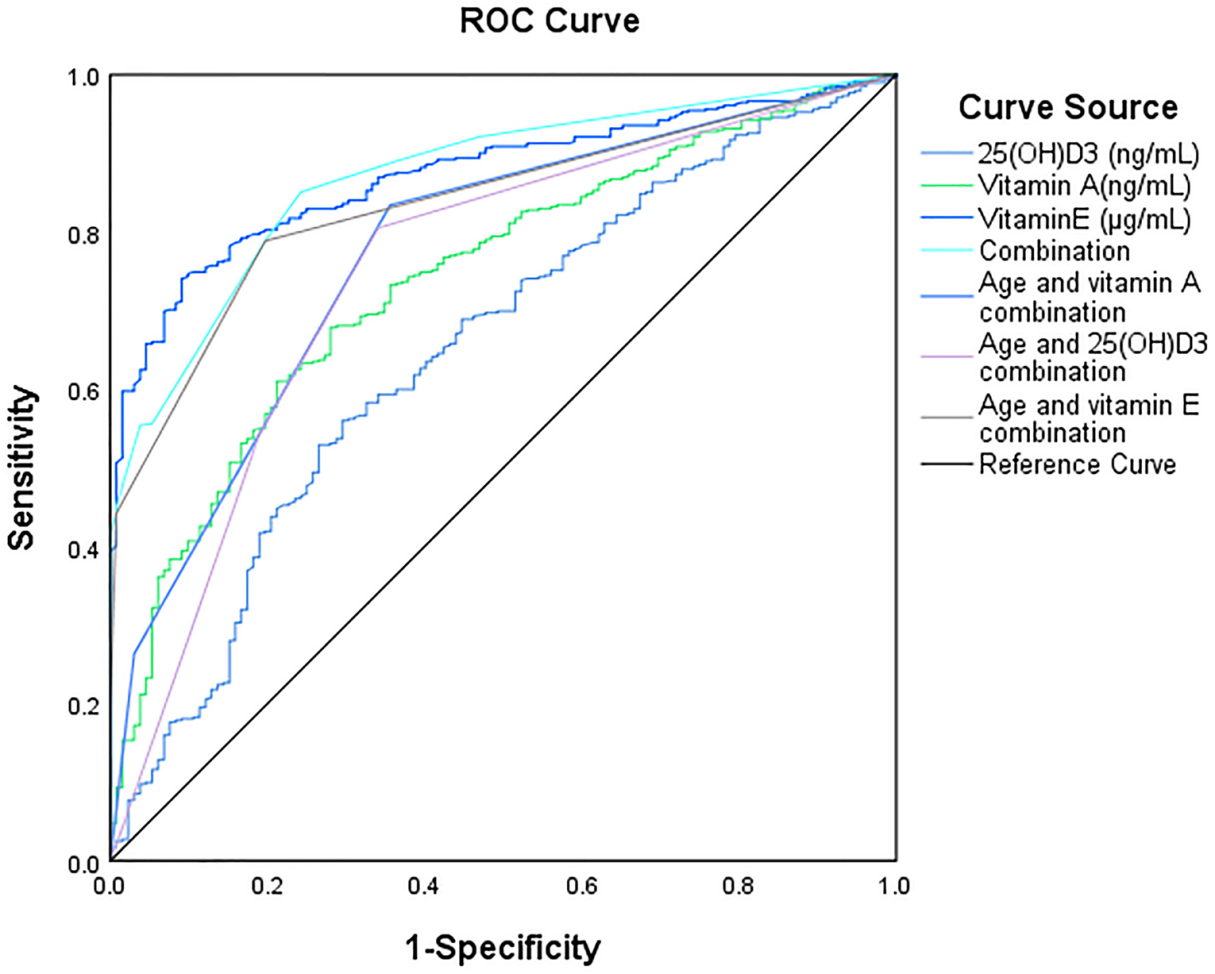
ROC curve for predicting growth retardation.

Meanwhile, we analyzed the relationship between different factors (age and vitamin level) and GR by Multivariable logistic regression analysis. The results revealed that age (OR = 0.104, 95% CI: 0.06–0.179, *p* < 0.01), Vitamin A (OR = 2.318, 95% CI: 1.232, 4.358, *p* < 0.05), Vitamin E (OR = 1.307, 95% CI: 1.001–1.869, *p* < 0.05), and 25(OH)D3 (OR = 2.802, 95% CI: 1.610–4.876, *p* < 0.05) were independent risk factors for GR (Table 3). The AUC of Vitamin E, Vitamin A,25(OH)D3, for predicting GR were 0.87, 0.74 and 0.65, respectively. However, the combination of Vitamin E, Vitamin A, 25(OH)D3 and age in predicting GR had an AUC of 0.88 (95% CI:0.84–0.90) (Figure 2, Table 4).

**Table 3:** Multivariable logistic regression analysis.

Index	OR	95% CI		*B*-value	*p*-value
		Lower limit	Superior limit		
Age	0.104	0.060	0.179	−2.265	<0.01
Vitamin A	2.318	1.232	4.358	0.841	<0.01
25(OH)D3	2.802	1.610	4.876	1.030	<0.01
Vitamin E	1.307	1.001	1.869	0.752	0.042
Vitamin K1	0.468	0.157	1.396	−0.759	0.173

CI, confidence interval; OR, odds ratio; B value: The regression coefficient and intercept.

**Table 4:** prediction performance.

Variable	AUC	*p*-value	95% CI		Cut off value	Sensitivity	Specificity	Youden index
			Lower limit	Superior limit				
Vitamin A	0.74	<0.01	0.70	0.79	252.45	0.68	0.72	0.40
25(OH)D3	0.65	<0.01	0.60	0.70	23.28	0.56	0.70	0.26
Vitamin E	0.87	<0.01	0.85	0.90	4.77	0.74	0.91	0.65
Age and vitamin A combination	0.77	<0.01	0.73	0.82	/	0.83	0.64	0.48
Age and 25(OH)D3 combination	0.75	<0.01	0.70	0.80	/	0.80	0.66	0.46
Age and vitamin E combination	0.84	<0.01	0.80	0.87	/	0.79	0.80	0.59
Combination	0.88	<0.01	0.84	0.90	/	0.85	0.76	0.61

CI, confidence interval; AUC, area under curve.

## Discussion

In this study, we examined the correlation between various factors (including maternal educational background, pregnancy-related diseases, gender, and age) and the incidence of children GR. Our findings revealed that GR in children is significantly associated with age, with the condition predominantly manifesting between 4 and 6 years of age. This observation aligns with the points of Nshimyiryoh (18) and Kismul (19). Children older than 3 years exhibit a notably higher risk of GR. Consequently, particular attention should be directed towards preschool children aged between 3 and 6, as this period represents a critical phase of rapid growth and development, particularly for bones and muscles (20).

**Figure 3:**
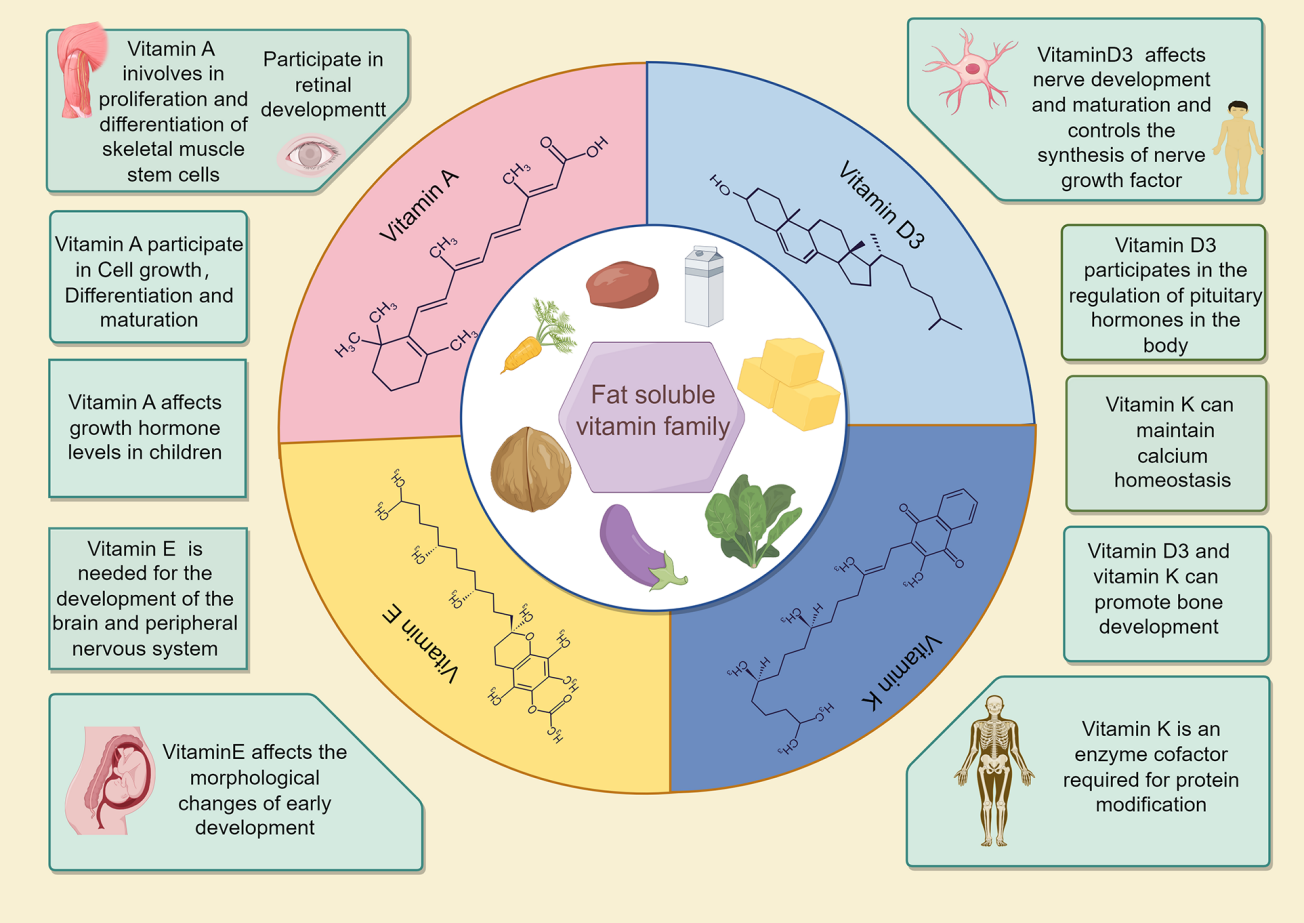
Effects of fat-soluble vitamins on body growth and development.

Physical growth and neural cell development are crucial for early childhood. Vitamins play a vital role in cell formation, bone growth, and other bodily functions for growth (21). Many fat-soluble vitamins, especially 25(OH)D, can regulate calcium and phosphorus metabolism (22–24) during bone and brain development (Figure 3) (25–27). According to an epidemiological survey, 1 billion people worldwide suffer from vitamin D deficiency or insufficiency (28, 29), and children in the growth stage are most vulnerable to its effects (30). The rate of 25(OH)D deficiency in children under 3 years of age is only 7.1%, which is 75% in children over 3 years of age (31). In this study, children with growth retardation had significantly lower 25(OH)D3 levels than those with normal development, reflecting the significance of 25(OH)D3 deficiency and its impact on growth retardation in children age 1–6 years.

Adequate amounts of vitamins A, E, and 25(OH)D3 are essential for growth promotion and protection. Adequate Vitamin A intake during delivery enhances the outcomes of newborns and promotes neural development in later childhood (32). Vitamin E deficiency can cause neural degeneration, atrophy, and purkinje cell death, leading to spinocerebellar ataxia, particularly during embryonic and early childhood neural development (Figure 3) (33–35). In this study, Vitamin A levels were significantly different between the two groups of children; Vitamin E levels were lower in the observation group. Combined with the results of Ernawati et al., we conclude that fat-soluble vitamin deficiency is not only the main cause of GR in children, but also may be the main reasons for the cognitive and neurodevelopmental delays observed in most of the children.

Despite the promising results and key findings, this study has some limitations. First, it was conducted in only one hospital, and the sample size was relatively small. Therefore, the findings of this study may not be applicable to other populations or settings. Second, the study only included children receiving treatment at the selected hospital, which may have led to selection bias. Third, the study did not control for potential confounding variables such as dietary intake, which could have influenced the findings.

## Data Availability

The original contributions presented in the study are included in the article/Supplementary Material, further inquiries can be directed to the corresponding author.
